# Lipid biomarker signatures as tracers for harmful cyanobacterial blooms in the Baltic Sea

**DOI:** 10.1371/journal.pone.0186360

**Published:** 2017-10-16

**Authors:** Thorsten Bauersachs, Helen M. Talbot, Frances Sidgwick, Kaarina Sivonen, Lorenz Schwark

**Affiliations:** 1 Department of Organic Geochemistry, Christian-Albrechts-University, Kiel, Germany; 2 School of Civil Engineering and Geosciences, Newcastle University, Newcastle upon Tyne, United Kingdom; 3 Department of Food and Environmental Sciences, University of Helsinki, Helsinki, Finland; 4 Department of Chemistry, Curtin University, Perth, Australia; Stazione Zoologica Anton Dohrn, ITALY

## Abstract

The recent proliferation of harmful cyanobacterial blooms (cyanoHABs) in the Baltic and other marginal seas poses a severe threat for the health of infested ecosystems as e.g. the massive export and decay of cyanobacterial biomass facilitates the spread of bottom water hypoxia. There is evidence that cyanoHABs occurred repeatedly in the Baltic Sea but knowledge of their spatiotemporal distribution and the cyanobacteria that contributed to them is limited. In this study, we examined representatives of the major bloom-forming heterocystous cyanobacteria (i.e. *Aphanizomenon*, *Dolichospermum* (formerly *Anabaena*) and *Nodularia*) to establish lipid fingerprints that allow tracking these environmentally important diazotrophs in the modern and past Baltic Sea. The distribution of normal and mid-chain branched alkanes, fatty acid methyl esters, bacteriohopanepolyols and heterocyst glycolipids permitted a clear chemotaxonomic separation of the different heterocystous cyanobacteria but also indicated a close phylogenetic relationship between representatives of the genera *Aphanizomenon* and *Dolichospermum*. Compared to the discontinuous nature of phytoplankton surveys studies, the distinct lipid profiles reported here will allow obtaining detailed spatiotemporal information on the frequency and intensity of Baltic Sea cyanoHABs as well as their community composition using the time-integrated biomarker signatures recorded in surface and subsurface sediments. As heterocystous cyanobacteria of the genera *Aphanizomenon*, *Dolichospermum* and *Nodularia* are generally known to form massive blooms in many brackish as well as lacustrine systems worldwide, the chemotaxonomic markers introduced in this study may allow investigating cyanoHABs in a great variety of contemporary environments from polar to tropical latitudes.

## Introduction

Massive accumulation of unicellular and/or filamentous cyanobacteria—known as harmful cyanobacterial blooms (cyanoHABs)—have significantly increased in abundance, intensity and duration in both brackish and freshwater environments over the last decades [1–3]. In the modern Baltic Sea, cyanoHABs are particularly frequent and during summer can cover large areas of surface water with profound impacts on the health of the aquatic ecosystem [4, 5]. This is because the decay of organic matter and its associated O_2_ consumption has resulted in a fourfold increase in the area affected by bottom water hypoxia and the spread of ecological ‘dead zones’ in the recent past [6, 7]. As a consequence, the modern Baltic Sea experiences a drastic loss of viable habitat of both benthic and pelagic faunal communities, as well as significant changes in nutrient cycling and ecosystem structure [8, 9]. The negative feedback on the Baltic Sea ecology is amplified by the fact that many bloom-forming cyanobacteria express the ability to synthesize strong hepato- and neurotoxins (e.g. anatoxin, microcystin, nodularin, or saxitoxin), which pose a significant health risk for both animals and humans and have led to the repeated poisoning of wildlife along the coasts of Sweden and Finland [10].

The major bloom-forming diazotrophs in the contemporary Baltic Sea belong to three genera of filamentous heterocystous cyanobacteria including *Aphanizomenon*, *Dolichospermum* (previously classified as *Anabaena*) and *Nodularia* [11, 12]. Representatives of these genera are considered important sources of combined N, as they add an estimated 180–420 Gg N yr^-1^ to the surface waters of the Baltic Sea, which is similar in magnitude to the entire riverine load and twice the atmospheric input [12, 13]. Long term monitoring programs indicate that the frequency and abundance of cyanoHABs have increased considerably since the early 1900s due to the massive anthropogenic loading of nutrients (in particular phosphorous) to the Baltic Sea [4, 14]. Given an undiminished high nutrient input, in combination with an anticipated increase in surface water temperature by 3–4°C until the end of the 21^st^ century [15], it has been predicted that cyanobacterial bloom-formation will be significantly enhanced in the future, along with intensification and greater frequency of hypoxia [9]. Current estimates on the future development of cyanobacterial blooms and their consequences for the Baltic Sea show, however, large uncertainties due to our incomplete understanding of the spatiotemporal distribution of cyanoHABs in the Baltic Sea over time and the anthropogenic as well as environmental controls that promote bloom formation.

Sediment-hosted lipid biomarkers constitute a powerful tool in ecosystem studies as they i) provide a time-integrated signal of the occurrence and composition of cyanoHABs and ii) allow characterizing the past occurrence and composition of such blooms in a high spatiotemporal resolution over geological timescales, provided that they can be linked unequivocally to their biological sources [16, 17]. Biomarkers considered to be characteristic for cyanobacteria and potentially suited for tracing cyanoHABs in modern and past ecosystems include mid-chain branched alkanes [18, 19], C-2 methylated bacteriohopanepolyols (BHPs) as well as their diagenetic products [20], and BHP structures (such as C-30,32,33,34,35-pentol BHP) only observed in cyanobacteria to date [21]. In addition, the polyunsaturated fatty acids (PUFAs) 18:2ω6, 18:3ω6 and 18:4ω3 have been reported in high abundances in certain genera of both unicellular as well as filamentous heterocystous cyanobacteria [22, 23]. These components are frequently used to investigate the composition of cyanobacterial communities in microbial mats [24] and extreme environments [25]. Heterocyst glycolipids (HGs) represent a novel suite of biomarkers that have been reported exclusively from the heterocyst cell envelope of heterocystous cyanobacteria and as such constitute the first and only biomarkers that allow tracing for the process of cyanobacterial N_2_ fixation in natural systems [26, 27]. Interestingly, the distribution of HGs has been shown to vary at least on a family level in cultured cyanobacteria [26, 27], so HGs may hold great promise for providing detailed information on the composition of cyanoHABs in aquatic ecosystems (such as the Baltic Sea) over time.

Although the morphology and molecular diversity of cyanobacteria in general and of heterocystous species in particular have been extensively studied in the past [28–32], there is surprisingly little comprehensive information on their biomarker inventory. Detailed lipid profiles, focusing mainly on cellular fatty acids and mid-chain branched alkanes, have been obtained from a few key genera, including among others *Anabaena*, *Nostoc*, *Synechocystis* or *Oscillatoria* [18, 22, 33, 34], but the great majority of cyanobacteria has yet only been inadequately studied. Furthermore, these investigations are largely biased towards freshwater cyanobacteria, whereas little is known about the lipid inventory of cyanobacteria growing in marine and brackish environments such as the Baltic Sea. In this study, we qualitatively assessed the lipid profiles of eight bloom-forming heterocystous cyanobacteria in order to (i) determine whether the distribution of specific lipids or individual lipid classes allows the chemotaxonomic separation of the studied genera and (ii) identify biomarkers suitable for tracing N_2_ fixing heterocystous cyanobacteria in the Baltic Sea and possibly other aquatic environments.

## Materials and methods

### Cyanobacterial cultures

Eight strains of heterocystous cyanobacteria belonging to the genera *Dolichospermum* (315, BIR53, BIR169), *Aphanizomenon* (TR183) and *Nodularia* (AV1, BY1, F81, HEM) had been isolated from the Baltic Sea and were obtained from the Culture Collection of Cyanobacteria (HAMBI) maintained at the Department of Food and Environmental Sciences at the University of Helsinki. Culture conditions are provided in S1 Table. Briefly, they were grown in 0.5 l Erlenmeyer flasks containing 0.2 l Z8X culture medium that was free of combined N and supplemented with NaCl at 8.75 g l^-1^ [35]. All strains were grown under continuous white fluorescent light at an irradiance of 8–9 μE m^-2^ s^-1^ and at a temperature of either 18°C (BIR53, BIR169, BY1, F81) or 23°C (315, TR183, AV1, HEM), respectively. All cultures were harvested towards the end of the logarithmic growth phase by centrifugation and stored frozen afterwards. Upon arrival at Christian-Albrechts-University, cells were lyophilized and kept at -20°C prior to solvent extraction. *Dolichospermum* BIR53 and BIR169 as well as *Nodularia* BY1 and F81 were non-axenic but on the basis of microscopy showed only low levels of bacterial contamination.

### Solvent extraction

Freeze dried biomass (ca. 80 mg) was extracted using a modified Bligh and Dyer technique [36]. Briefly, the cell material was extracted ultrasonically 3x for 10 min in a mixture of MeOH, dichloromethane (DCM) and phosphate buffer (2:1:0.8; v/v/v). After each sonication step, the mixture was centrifuged at 1,500 × g for 6 min and the supernatant transferred to a centrifuge tube. The combined supernatants were phase separated by adding DCM and phosphate buffer to a final solvent ratio of 1:1:0.9 (v/v/v). The organic bottom layer was transferred to a round bottom flask and reduced under vacuum using a rotary evaporator. Each Bligh and Dyer extract (BDE) was transferred to a pre-weighed glass vial using DCM:MeOH (9:1; v/v) and dried under a gentle stream of N_2_.

### Hydrocarbon analysis

An aliquot of the BDE (5–6 mg) was separated into an apolar and a polar fraction using activated Al_2_O_3_ as stationary phase and *n*-hexane:DCM (9:1; v/v) and DCM:MeOH (1:1; v/v) as respective eluents. The apolar fraction, containing aliphatic hydrocarbons, was dried under a gentle stream of N_2_ and dissolved in *n*-hexane to a concentration of 1 mg ml^-1^ prior to analysis. Gas chromatography-mass spectrometry (GC-MS) was performed using an Agilent 7820A gas chromatograph equipped with a Phenomenex ZB-5 fused silica column (30 m × 0.25 mm i.d.; 0.25 μm film thickness). Helium (He) was used as carrier gas at a constant 1 ml min^-1^. The injector was at 70°C and the oven programmed to 130°C at 20°C min^-1^ and then at 4°C min^-1^ to 320°C (held 10 min). The GC instrument was interfaced with an Agilent 5975 MSD mass spectrometer operated at 70 eV and scanning *m/z* 50 to 650 with a cycle time of 1.7 s (resolution 1000). Straight chain and branched alkanes were assigned from retention time indices as described in Kissin et al. [37] and comparison with published mass spectra [38, 39].

### Fatty acid methyl ester analysis

Freeze-dried and homogenized cell material (3 mg) was extracted and transesterfied as detailed in Mudimu et al. [40]. The extract was dissolved in DCM to a concentration of 0.5 mg ml^-l^ and subjected to the analysis of FAMEs using an Agilent 7820A gas chromatograph equipped with a Phenomenex ZB-Wax column (30 m × 0.25 mm i.d.; 0.50 μm film thickness) and connected to a flame ionization detector (FID). Hydrogen was the carrier gas at 1 ml min^-1^. The oven temperature was programmed from 50°C (1 min) to 200°C at 25°C min^-1^, then to 230°C (held 18 min) at 3°C min^-1^. FAMEs were assigned by comparison of retention times with those of the “Supelco 37 Component FAME mix” (Sigma-Aldrich, Germany).

FAMEs not included in the standard mixture were assigned from their mass spectral characteristics using an Agilent 7820A gas chromatograph coupled to an Agilent 5975 MSD mass spectrometer. Analytical conditions were as above. Assignment of FAMEs was accomplished by comparison of mass spectra with those in the “Lipid Library” of the American Oil Chemists’ Society (http://lipidlibrary.aocs.org).

### Heterocyst glycolipid analysis

An aliquot of the BDE (ca. 1 mg) was dissolved in DCM:MeOH (9:1, v/v) to a concentration of 0.5 mg ml^-1^ and filtered through a 0.45 μm regenerated cellulose syringe filter. Heterocyst glycolipids were analyzed as described by Bauersachs et al. [41] with some modifications. Briefly, normal phase high performance liquid chromatography (HPLC) was carried out using a Waters Alliance 2690 HPLC system fitted with a Phenomenex Luna NH_2_ column (150 × 2 mm; 3 μm particle size) and a guard column of the same material, which both were maintained at 30°C. Separation of HGs was accomplished with the following gradient profile: 95% A/5% B to 85% A/15% B in 10 min (held 7 min) at 0.5 ml min^-1^, followed by back flushing with 30% A/70% B at 0.2 mg ml^-1^ for 25 min and re-equilibrating the column with 95% A/5% B for 15 min. Solvent A was *n*-hexane:2-propanol:HCO_2_H:14.8 M NH_3_ aq. (79:20:0.12:0.04; v/v/v/v) and solvent B was 2-propanol:water:HCO_2_H:14.8 M NH_3_ aq. (88:10:0.12:0.04; v/v/v/v). All solvents and additives were at least of HPLC-grade.

Detection of HGs was performed using a Quattro LC triple quadrupole mass spectrometer (Micromass, UK) equipped with an electrospray ionization (ESI) interface and operated in positive ion mode. Source conditions were: capillary 3.5 kV, cone 20 V, desolvation temperature 230°C, source 120°C; cone gas 80 l h^-1^ and desolvation gas 230 l h^-1^. BDEs were initially screened in data-dependent mode with two scan events, where a scan of *m/z* 300–1000 was followed by a product ion scan of the base peak in the spectrum of the first scan event. Assignment of HGs was achieved by comparison of mass spectra published in the literature [42, 43]. As some of the eight identified target compounds occurred only in low abundance, all cultures were re-analyzed using multiple reaction monitoring (MRM) to increase sensitivity. MRM experiments were performed as outlined by Bauersachs et al. [44] with transitions monitored as follows: *m/z* 547 → 415 (pentose HG_26_ diol), *m/z* 561 → 415 (deoxyhexose HG_26_ diol), *m/z* 575 → 413 (HG_26_ keto-ol), *m/z* 577 → 415 (HG_26_ diol), *m/z* 603 → 441 (HG_28_ keto-ol), *m/z* 605 → 443 (HG_28_ diol), *m/z* 619 → 457 (HG_28_ keto-diol) and *m/z* 621 → 459 (HG_28_ triol). HGs of higher chain length were not detected in the investigated Baltic Sea cyanobacteria.

As the structure of the HG backbones cannot be unequivocally determined by HPLC/MS alone, cell material of each heterocystous cyanobacterium was acid hydrolyzed and analyzed following the procedure detailed in Bauersachs et al. [42]. Briefly, freeze-dried biomass (~30 mg) of each cyanobacterium was suspended in a mixture of MeOH:10.2M hydrochloric acid (95:5; v/v) and refluxed for 3 h at 80°C to cleave the aglycone moieties from their sugar head groups. HPLC-grade water was then added and the reaction products were extracted thrice with DCM:MeOH (2:1; v/v). The organic bottom layer was transferred to a round bottom flask and the remaining aqueous phase extracted twice with DCM. Subsequently, the hydrolyzed total lipid extracts were reduced under vacuum and dried under a gentle stream of N_2_. The liberated polyhydroxyalcohols were converted to trimethylsilyl ethers by heating at 60°C for 2 h with 25 μl of each bis(trimethylsilyl)trifluoroacetamide (BSTFA) and pyridine prior to GC-MS analysis.

The derivatized polyhydroxyalcohols were analysed using an Agilent 7820A gas chromatograph fitted with a Phenomenex ZB-5 fused silica column (30 m × 0.25 mm i.d.; 0.25 μm film thickness). Helium (He) was used as carrier gas at a constant 1 ml min^-1^. The injector was at 70°C and the oven programmed to 130°C at 20°C min^-1^ and then at 5°C min^-1^ to 320°C (held 20 min). The GC instrument was interfaced with an Agilent 5975 MSD mass spectrometer operated at 70 eV and scanning *m/z* 50 to 850 with a cycle time of 1.7 s (resolution 1000). Identification of HG degradation products was performed by comparison with published mass spectra [42, 45].

### Bacteriohopanepolyol analysis

Analysis of BHPs was carried out at Newcastle University. Freeze-dried biomass (ca. 80 mg) was extracted using a modified Bligh and Dyer technique as described previously [46]. In brief, the biomass was weighed into a Teflon centrifuge tube and extracted using 19 ml of a monophasic mixture of MeOH, chloroform (CHCl_3_) and H_2_O (10:5:4; v/v/v). The mixture was sonicated (40°C, 10 min) and centrifuged at 17,200 × g for 15 min. The supernatant was then transferred to a new centrifuge tube and the residue was re-extracted twice using the same procedure. The tube containing the supernatant was phase-separated by adding 5 ml of each CHCl_3_ and H_2_O and subsequent centrifugation at 17,200 × g for 5 min. The organic bottom layer was rotary evaporated to near dryness and transferred to a pre-weighed glass vial using CHCl_3_:MeOH (2:1; v/v) and dried under a gentle stream of N_2_. An aliquot of the BDE (ca. 6 mg) was acetylated by heating with 0.5 ml acetic anhydride (Ac_2_O):pyridine (1:1; v/v) at 50°C for 1 h and letting it react at room temperature overnight. The derivatization agents were removed under N_2_ and the acetylated TLE dissolved in 0.5 ml MeOH:2-propanol (3:2; v/v) prior to analysis.

Analysis of BHPs was achieved using reversed phase HPLC-atmospheric pressure chemical ionization mass spectrometry (HPLC-APCI-MS^n^) with a ThermoFinnigan surveyor HPLC system equipped with a Phenomenex Gemini C_18_ column (150 × 3.0 mm, 5 μm particle size) and a security guard column cartridge of the same material, coupled to a Finnigan LCQ ion trap mass spectrometer. BHPs were eluted at a constant 30°C and using the following gradient: 90% A/10% B (0 min) to 59% A/1% B/ 40% C (at 25 min), then isocratic to 45 min, returning to the starting conditions in 5 min and stabilizing for 10 min with A = MeOH, B = H_2_O, C = 2-propanol. Solvent flow was 0.5 ml min^-1^. The mass spectrometer was equipped with an APCI source operating in positive ionization mode with the following settings: capillary 150°C, APCI vaporizer 490°C, corona discharge 8 μA, sheath gas flow 40 and auxiliary gas flow of 10. MS^n^ analysis was carried out in data dependent mode with 2 scan events. Scan event 1 was a full MS scan from *m/z* 300–1300. Scan event 2 was data dependent and utilized the most intense ions from scan event 1 to perform MS^2^ scans. Assignment of BHPs was achieved by comparison of mass spectra with those published in the literature [46, 47].

### Statistical analysis

Correspondence analysis (CA) was performed to investigate similarities/differences in whole lipid profiles and distribution pattern of individual lipid classes using XLSTAT (v. 18.06) software package for Microsoft Excel. The axes of the CA plots essentially measure the amount of variation from the average, with the most common lipids located closest to the origin and those being highly specific for a certain species or genera visualizing at the plot extremes [48]. The axes of the graphs were chosen to show the largest fraction of variability in the multidimensional data matrix. Thus, the most informative axes are in the direction of maximum variance of the scatter of points. The inertia value of an axis is the proportion of variance explained by it.

## Results and discussion

### Straight chain and branched hydrocarbons

**Fig 1:**
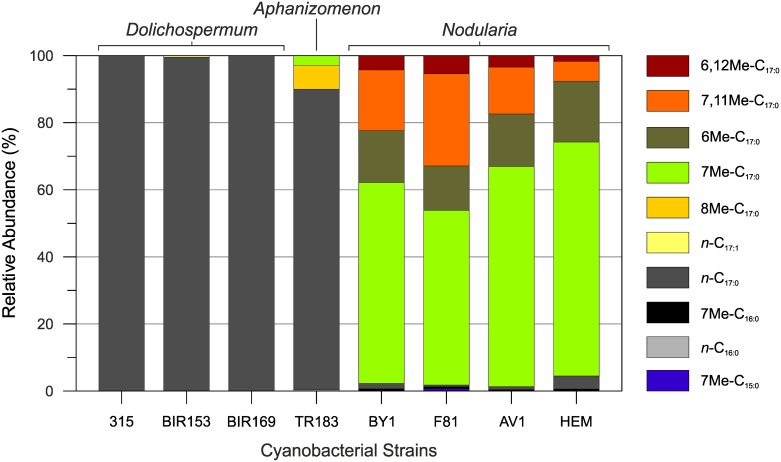
Distribution of hydrocarbons in the Baltic Sea cyanobacteria *Dolichospermum*, *Aphanizomenon* and *Nodularia*.

The hydrocarbons included straight chain as well as mono- and dimethyl alkanes with 16 to 19 carbons and distinct distribution patterns among the genera (Fig 1; S2 Table). In species belonging to *Dolichospermum* (formerly *Anabaena*), *n*-heptadecane (*n*-C_17:0_) was the only hydrocarbon detected, with the exception of strain BIR53, in which traces (<1%) of *n*-hexadecane (*n*-C_16:0_) and *n*-heptadecene (*n*-C_17:1_) were also present. These results are well in line with the previously reported hydrocarbon distribution in freshwater representatives of this genus [34]. *n*-Heptadecane has been reported to be among the most prominent hydrocarbons in various cultured unicellular and filamentous heterocystous cyanobacteria [49–51]. It also occurred in high abundances in hypersaline microbial mats [52] as well as hot springs [53, 54] and in such extreme environments is generally considered to be of cyanobacterial origin. However, *n*-C_17:0_ is present in many aquatic algae and photosynthetic bacteria [55] and in non-extreme environments it may not serve as an indicator for a cyanobacterial contribution to the sedimentary organic matter. Biological markers that are more specific for a cyanobacterial origin are mid-chain branched mono-, di-, and trimethylalkanes [18, 49, 56]. These components were not detected in any of the investigated *Dolichospermum* strains, although 7-methylheptadecane (7Me-C_17:0_) and 8-methylheptadecane (8Me-C_17:0_) have been reported in a representative of this genus before [34, 57]. None of the *Dolichospermum* strains isolated from the Baltic Sea seems, however, to be a potent source of mid-chain branched alkanes.

Strain TR183 was the only heterocystous cyanobacterium of the genus *Aphanizomenon* investigated here and to the best of our knowledge is the first representative of this genus analyzed for its hydrocarbon composition. It contained *n*-C_17:0_ as the most dominant hydrocarbon (89.7%) and traces of *n*-C_16:0_ (0.3%; S2 Table). Small quantities of monomethyl alkanes (MMAs; 10.0%) were also detected with 8Me-C_17:0_ the most dominant (7.1%) followed by 7Me-C_17:0_ (2.9%). Interestingly, 8Me-C_17:0_ was not found in any of the other heterocystous cyanobacteria and it may thus be of chemotaxonomic value in paleoenvironmental studies addressing the role of N_2_-fixing heterocystous cyanobacteria in the Baltic Sea. It should be noted, however, that 8Me-C_17:0_ and 7Me-C_17:0_ constitute the most frequently reported MMAs from extant cyanobacteria, including both unicellular and filamentous species [34, 58], and at this point it cannot be excluded that diazotrophs other than *Aphanizomenon* TR183 may provide a source of 8Me-C_17:0_ in the Baltic Sea.

The apolar fraction of *Nodularia* spp. contained only a low relative proportion of *n*-C_17:0_ (0.6 to 3.9%) but abundant MMAs and dimethyl alkanes (DMAs) that constituted >95% of all hydrocarbons. The major MMAs were 7Me-C_17:0_ and 6-methylheptadecane (6Me-C_17:0_), which together accounted for 65.3 to 87.8% of all hydrocarbons, respectively (Fig 1; S2 Table). We also observed traces (<1%) of 7-methylhexadecane (7Me-C_16:0_) in all strains and 7-methylpentadecane (7Me-C_15:0_) exclusively in *Nodularia* F81. A unique feature of *Nodularia* spp. was the presence of DMAs that occurred in comparatively high abundance (7.6 to 32.9%; S2 Table). The 7,11Me-C_17:0_ was usually dominant, ranging from 5.9 to 27.5%, while 6,12Me-C_17:0_ occurred only in small quantity (<5%). DMAs have been reported from some pure cultures of filamentous cyanobacteria including *Calothrix scopulorum* [56] and *Phormidium luridum* [19] but they usually occur in low abundance and, compared with MMAs, seem to be less widespread in cyanobacteria. However, they are frequently recognized in modern coastal and hot spring microbial mats, in which cyanobacteria represent a major constituent of the microbial community [39, 53, 54] and in the Baltic Sea they may represent valuable biomarkers for tracing heterocystous cyanobacteria of the genus *Nodularia*.

In addition to the suite of straight-chain and branched alkanes and alkenes, we also detected the pentacyclic triterpenoid hop-22(29)-ene (diploptene) in the hydrocarbon fraction of all *Dolichospermum* and *Aphanizomenon* strains. This component has been reported from a vast number of cultured unicellular and filamentous cyanobacteria [18, 59] and it is also known to occur ubiquitously in modern hot spring microbial mats, where it is commonly attributed to a cyanobacterial origin [58, 60]. However, it should be pointed out that it is widely distributed within the bacterial domain [59] and has been reported, among others, from sulfate reducing bacteria [61] as well as methanotrophic bacteria [62]. It is interesting to note that diploptene and bacteriohopanepolyols (see below) were not detected in any of the four *Nodularia* strains because both are considered essential in regulating membrane rigidity and permeability in the prokaryotic cell [63]. Our results are, however, in agreement with the observation that <10% of all prokaryotes may in fact synthesize hopanoid structures [64], which raises the questions about whether components other than hopanoids regulate the fluid transport across the cell membrane of *Nodularia* spp. or whether these components are indeed essential for the biology of the prokaryotic cell.

### Saturated, monounsaturated and polyunsaturated fatty acids

Fatty acids (FA) constitute a major component of the bacterial and eukaryotic cell membrane. They are ubiquitously present in sedimentary environments, where their characteristic distribution patterns are studied to obtain information on the structure and composition of modern and fossil microbial communities [65]. Pure cultures of cyanobacteria are generally characterized by relatively simple cellular FA distributions dominated by 16:0, 16:1ω9, 18:2ω6 or 18:3ω3 [33, 66, 67]. The dominance of these components in e.g. microbial mats [24, 68] is usually taken as evidence for the presence of cyanobacteria; in particular in extreme settings where the harsh environmental conditions limit the activity of eukaryotes [25].

**Fig 2:**
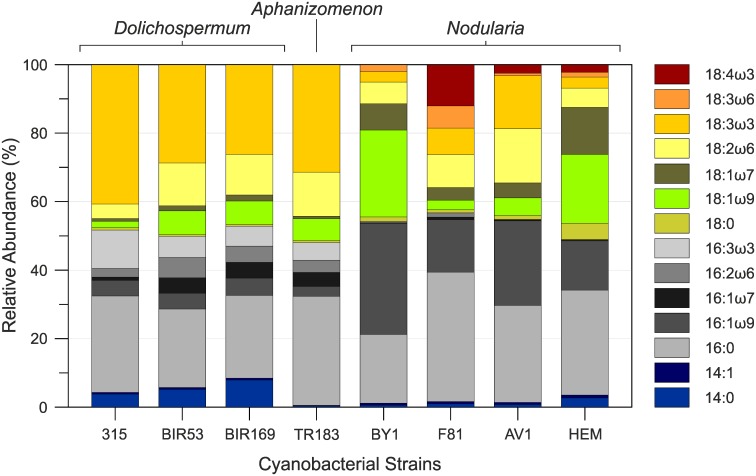
Distribution of FAMEs in Baltic Sea cyanobacteria belonging to the genera *Dolichospermum*, *Aphanizomenon* and *Nodularia*.

Here, 16:0 (25.1±2.7%), 18:2ω6 (9.5±4.6%) and 18:3ω3 (31.9±7.8%) were the dominant FAMEs in all of the *Dolichospermum* strains (Fig 2; S3 Table). Other FAMEs occurring in lower abundance included 14:0 (5.6±2.1%), 16:1ω9 (4.8±0.3%), 16:3ω3 (7.7±3.0%) and 18:1ω9 (5.2±2.9%). Taken together, these seven components accounted for the majority of the identified FAMEs (≥80%). A common characteristic of all *Dolichospermum* strains was the comparatively high content of polyunsaturated fatty acids (PUFAs), which accounted for 53.5±5.1% of the total FAMEs. Saturated fatty acids (SAFAs) and monounsaturated fatty acids (MUFAs) each occurred in significantly lower abundance, constituting 31.3±2.3% and 15.2±5.7%, respectively (S3 Table). In terms of distribution and relative abundance, the FAME content of our *Dolichospermum* strains is thus in good agreement with those previously reported for freshwater representatives of this genus [23, 33, 69] and isolates obtained from the Baltic Sea [67, 70]. All *Dolichospermum* strains exclusively contained higher relative proportions of 14:0, which occurred in trace amount only in the *Aphanizomenon* and *Nodularia* strains (<1%) with the exception of *Nodularia* HEM, where it constituted 2.7% (S3 Table). In a previous study on the FAME content of Baltic Sea cyanobacteria, 14:0 was found to be abundant only in hepatotoxic *Dolichospermum* (previously defined as *Anabaena*) strains [67]. Although this finding is not supported by our study, as both non-toxic (BIR53 and BIR169) and hepatotoxic (315) *Dolichospermum* strains contained a similar proportion of 14:0, its generally low abundance in the other cyanobacterial genera investigated here suggests that it is of chemotaxonomic relevance and may be suited to tracing the presence of *Dolichospermum* spp. in Baltic Sea surface waters and sediments.

The FAME profile of *Aphanizomenon* TR183 was dominated by 16:0 (31.9%), 18:2ω6 (12.9%) and 18:3ω3 (31.4%) which, taken together, constituted > 76.2% of all FAMEs. In addition, we detected 16:1ω7 (4.1%), 16:3ω3 (5.2%) and 18:1ω9 (6.4%) in moderate abundances and low or trace amounts of 14:1ω9 (0.2%), 16:1ω9 (2.9%), 16:2ω6 (3.6%), 18:0 (0.6%) and 18:1ω7 (0.7%). PUFAs constituted 53.0%, while SAFAs and MUFAs accounted for 32.8% and 14.2%, respectively (S3 Table). The FAME profile thus resembles those reported for *Aphanizomenon* TR183 [67] and other representative of this genus isolated from the Baltic Sea [70]. Comparison of the distribution pattern and relative abundance of individual FAMEs in *Aphanizomenon* TR183 and the three *Dolichospermum* strains showed an overall strong similarity, except for the virtual absence of 14:0 from *Aphanizomenon* TR183, suggesting a close phylogenetic relationship between both genera. This result is generally in line with data obtained from the analysis of 16S rRNA sequences, showing that species of *Aphanizomenon* and *Dolichospermum* occur interspersed in phylogenetic trees [32, 69, 71] and that *Aphanizomenon* TR183 clusters together with neurotoxic *Dolichospermum* strains isolated from Finnish freshwater lakes [30]. As only one strain of the genus *Aphanizomenon* was investigated here, it is of course difficult to generalize our results. However, molecular analysis suggests that the genetic diversity in *Aphanizomenon* is low, with only one phenotype dominating in the Baltic Sea [72, 73]. Hence, FAME profiles for representatives of the genus *Aphanizomenon* are expected to show little intraspecies variation. Such a perception is in line with the analysis of FAME profiles in five planktonic *Aphanizomenon* strains (including TR183) that in general show a very similar distribution pattern [67].

In contrast, heterocystous cyanobacteria of the genus *Nodularia* expressed FAME contents distinctly different from those in *Dolichospermum* spp. and *Aphanizomenon* sp. (Fig 2; S3 Table). The dominant FAMEs were 16:0 (29.3±7.4%), 16:1ω9 (21.8±8.5%) and 18:1ω9 (13.4±11.1%). Other FAMEs of significance included 18:1ω7 (7.4±4.6%) and 18:2ω6 (9.4±4.7%). In accord with this observation, MUFAs (43.5±18.5%) dominated the profile, followed by SAFAs (32.6±8.2%) and PUFAs (24.0±13.6%). Although generally present in low abundances (with the exception of *Nodularia* F81), the PUFAs 18:3ω6 (2.7±2.6%) and 18:4ω3 (4.0±5.1%) may be particularly interesting for chemotaxonomic studies as they were not detected in the other two genera of heterocystous cyanobacteria (Fig 2). Both components have been described from the freshwater dwelling *Nodularia sphaerocarpa* [74] and *Nodularia spumigena* isolated from the Baltic Sea [70], suggesting that they are a common component of the FAME content of the genus *Nodularia*. Given that 18:3ω6 and 18:4ω3 are less frequently observed in other microalgal classes, despite the sporadic occurrence of 18:4ω3 in some genera of eukaryotic algae [74], both components may be particularly diagnostic for heterocystous cyanobacteria of the genus *Nodularia* in the Baltic Sea.

Based on variation in FA content, cyanobacteria are currently divided in four distinct groups [33, 75]. The first group is devoid of PUFAs and contains only SAFAs and MUFAs. The second and third groups are characterized either by the presence of 18:3ω3 or 18:3ω6, respectively, while the fourth also has 18:4ω3. According to this classification, all *Dolichospermum* and *Aphanizomenon* strains investigated here can be assigned to group 2. On the contrary, heterocystous cyanobacteria belonging to the genus *Nodularia* fall within group 4 of the Kenyon-Murata classification scheme. Hence, compositional differences in the FAME distribution pattern between bloom-forming Baltic Sea cyanobacteria allow a clear separation of representatives of the genus *Nodularia* from the genera *Dolichospermum* and *Aphanizomenon* and again suggest a close phylogenetic relationship between the latter two genera.

### Heterocyst glycolipids

Heterocyst glycolipids occur exclusively in the heterocyst cell envelope of N_2_-fixing heterocystous cyanobacteria [76], where they are considered to act as a gas diffusion barrier that limits the entry of atmospheric gases into the heterocyst [77]. The structure of HGs consists of a sugar head group (e.g. hexose, deoxyhexose or pentose) glycosidically bound to an even numbered aglycone moiety with 26 to 32 carbons and functional groups (as OH and/or C = O moieties) attached to the C-3, ω-1 and ω-3 position [26, 42]. Investigations of the distribution of HGs in pure cultures of heterocystous cyanobacteria indicated that the chain length varies between different orders and families, with e.g. 1-(O-hexose)-3,25-hexacosanediols (HG_26_ diols) and 1-(O-hexose)-3-keto-25-hexacosanols (HG_26_ keto-ols) dominating in Nostocaceae and 1-(O-hexose)-3,27,29-triacontanetriols (HG_30_ triols) and 1-(O-hexose)-3-keto-27,29-octacosanediols (HG_30_ keto-diols) being representative for members of the Scytonemataceae [26, 27]. This genetically controlled variation in the structural composition of the heterocyst cell envelope has been suggested to offer the possibility of detailed investigations of cyanobacterial community compositions in natural environments [42] and may be of particular relevance in studying the composition of modern and past Baltic Sea cyanobacterial blooms.

**Fig 3:**
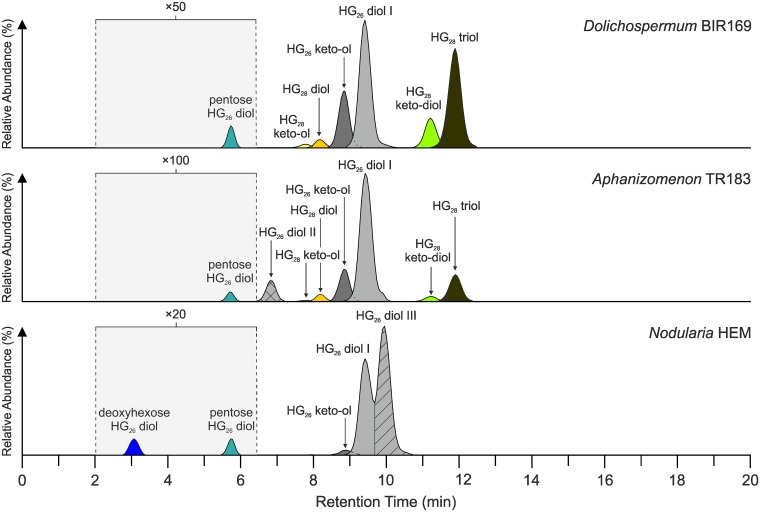
Composite mass chromatograms showing the distribution of heterocyst glycolipids (HGs) in Baltic Sea cyanobacteria.

**Fig 4:**
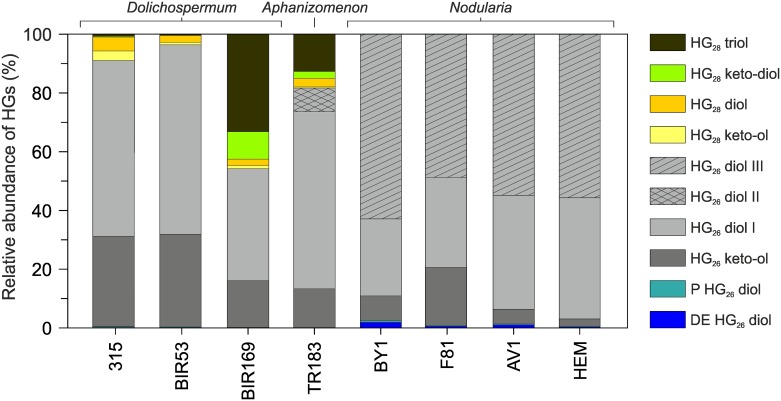
Relative abundance of heterocyst glycolipids (HGs) in Baltic Sea cyanobacteria.

HG distribution patterns for representatives of the three genera of Baltic Sea cyanobacteria are shown in Fig 3. All *Dolichospermum* strains were dominated by HG_26_ diol I (55.3±14.1%) and HG_26_ keto-ol (26.2±8.9%) that in general comprised >90% of all HGs with the exception of *Dolichospermum* BIR169, in which both components constituted no more than 54.1% (Fig 4; S4 Table). 1-(O-hexose)-3,27-octacosanediol (HG_28_ diol; 3.1±1.5%) and 1-(O-hexose)-3-keto-27-octacosanol (HG_28_ keto-ol; 1.7±1.4%) comprised the second most abundant group of HGs that on average amounted to 4.8±2.8% of the total HG pool. 1-(O-hexose)-3,25,27-octacosanetriol (HG_28_ triol; 0.5±0.3%) and 1-(O-hexose)-3-keto-25,27-octacosanediol (HG_28_ keto-diol; 0.2±0.2%) usually occurred in traces in *Dolichospermum* 315 and *Dolichospermum* BIR53 isolated from the Gulf of Finland, but in *Dolichospermum* BIR169 obtained from the central Baltic Proper [32], both components contributed significantly to the HG content (42.6%). The comparatively high proportions of HG_28_ triol and HG_28_ keto-diol in *Dolichospermum* BIR169 is interesting given that both components have to date been reported in major abundances only from representatives of the genus *Calothrix* (Rivulariaceae) [27] and the freshwater cyanobacterium *Cylindrospermopsis raciborskii* (Nostocaceae) [43]. In the Baltic Sea, HG_28_ triol and HG_28_ keto-diol have been found in surface sediments of the Landsort Deep [78] but their biological origin was elusive. Given that species belonging to *Calothrix* have only been described from benthic microbial mats of the Gulf of Finland [10], planktonic heterocystous cyanobacteria of the genus *Dolichospermum* may represent a potent source of HG_28_ triol and HG_28_ keto-diol in the Baltic Proper. In addition, we also detected traces of a HG_26_ diol with a pentose instead of the more commonly reported hexose head group (pentose HG_26_ diol) in all *Dolichospermum* strains (0.2±0.1%). This component has only been reported from *Aphanizomenon ovalisporum* and *C*. *raciborskii* so far [43]. Its subordinate presence in the Baltic Sea *Dolichospermum* strains as well as in representatives of the other genera investigated here (see below) suggests, however, that it may be more widespread among heterocystous cyanobacteria.

*Aphanizomenon* TR183 showed a HG distribution that similar to the other investigated component classes revealed a strong resemblance to those observed in the *Dolichospermum* strains (Fig 4). Two structural isomers of the HG_26_ diol (together comprising 68.2%) and HG_26_ keto-ol (13.2%) were the most dominant components, constituting 81.6% of all HGs (S4 Table). In addition to HG_26_ diol (60.1%) eluting at 9.3 min, we observed an early eluting structural isomer (HG_26_ diol II; 8.1%) at 6.8 min that has been reported from an axenic culture of *Anabaena* CCY9613 [44] and water column filtrates taken from a small freshwater lake in northern Germany [79]. Acid hydrolysis of cell material of *Aphanizomenon* TR183 released high amounts of a polyhydroxyalcohol with MS characteristics corresponding to those reported for 1,3,25-hexacosanetriol [42, 45], the degradation product of HG_26_ diol. Structural isomers of this component were not detected, suggesting that both HG_26_ diols in *Aphanizomenon* TR183 differed in the type of head group attached to the aglycone moiety. Indeed, the composition and configuration of the sugar functionality in heterocystous cyanobacteria is known to be highly variable, with α- and β-glucosides usually dominating over α-mannosides and/or α-galactosides [26]. Other HGs detected in *Aphanizomenon* TR183 included small quantities of HG_28_ diol (2.9%) and traces of HG_28_ keto-ol (0.4%) together with pentose HG_26_ diol (0.3%). The suite of HGs was completed by the presence of comparatively high relative abundances of HG_28_ triol (12.7%) and keto-diol (2.3%), constituting 15.0% to all HGs. Although both components occurred in lower proportions than in *Dolichospermum* BIR169, it seems plausible that *Aphanizomenon* TR183 constitutes a second important biological source of HG_28_ triol and HG_28_ keto-diol in the Baltic Sea. In fact, the HG distribution pattern as well as the relative abundance of individual HGs in *Aphanizomenon* TR183 closely resemble those reported from surface sediments of the Landsort Deep [78]. This strong similarity suggests that *Aphanizomenon* TR183 may be a major bloom former in the Baltic Sea, in full agreement with phylogenetic analysis and studies of the species composition of cyanoHABs in the modern Baltic Sea [10, 80].

All *Nodularia* strains expressed a comparatively simple HG distribution pattern with only four different types of HGs present. HG_26_ diols (89.8±12.8%) and HG_26_ keto-ol (9.0±7.7%) were again the most abundant HGs, which together accounted for 97.7 to 99.4% of all HGs (Fig 4; S4 Table). Such a predominance of HG_26_ diols and HG_26_ keto-ol has been observed before in *Nodularia chucula* and other representatives of this genus [27]. A unique characteristic of *Nodularia* spp. was the presence of two structural isomers of the HG_26_ diol, of which the latter eluting isomer (HG_26_ diol III; eluting around 10.1 min) was not detected in any of the other Baltic Sea cyanobacteria. In *Nodularia* spp., however, it was generally the most abundant of all HGs, ranging from 48.8 to 62.9% (55.6±5.8%). GC-MS analysis of the total lipid extract liberated after acid hydrolysis of cell material of *Nodularia* BY1 revealed the presence of large quantities of the same polyhydroxyalcohol observed in *Aphanizomenon* TR183. Structural isomers of 1,3,25-hexacosanetriol were, however, again not detected, indicating that both HG_26_ diol isomers differed in the composition and/or configuration of their sugar moiety. Although the exact structure of HG_26_ diol III remains to be determined, it may hold promise for tracing heterocystous cyanobacteria of the genus *Nodularia* in surface waters and sediments of the Baltic Sea, as it was absent from the other cyanobacteria investigated. In addition, pentose HG_26_ diol was detected in all *Nodularia* strains in trace amount (0.3±0.1%). All representatives of the genus *Nodularia* also contained small quantities of 1-(O-deoxyhexose)-3,25-hexacosanediol (deoxyhexose HG_26_ diol) that constituted up to 1.8% of all HGs in *Nodularia* BY1 but which was not detected in the other Baltic Sea cyanobacteria. At present, there exists little information on the distribution of this component in heterocystous cyanobacteria. It was for the first and only time reported from *A*. *aphanizomenoides* and *C*. *raciborskii* [43]. Given that deoxyhexose HG_26_ diol thus occurs in at least three different genera, it may be more widespread among heterocystous cyanobacteria but further cultures studies are necessary to fully elucidate the chemotaxonomic potential of this component.

### Bacteriohopanepolyols

Bacteriohopanepolyols are highly functionalized pentacyclic triterpenoids present in a wide range of prokaryotes [59]. Due to the large diversity of BHP structures in pure cultures of bacteria and, given their high taxonomic value, these components offer potential for fingerprinting bacterial communities and processes in modern and fossil ecosystems [81]. In cyanobacteria, the most frequently reported BHPs include bacteriohopanetetrol (BHT), bacteriohopanepentol (BHpentol) and composite BHPs such as BHT cyclitol ether [21]. BHPs with an additional methyl group at C-2 are considered most diagnostic for cyanobacteria [20], although only selected unicellular and filamentous cyanobacteria indeed synthesize 2Me-BHPs [21] and that C-2 methylation also occurs in other prokaryotes including methylotrophs [82], N_2_-fixing bacteria [83] and two strains of the purple non-sulfur bacterium *Rhodopseudomonas palustris* [84].

**Fig 5:**
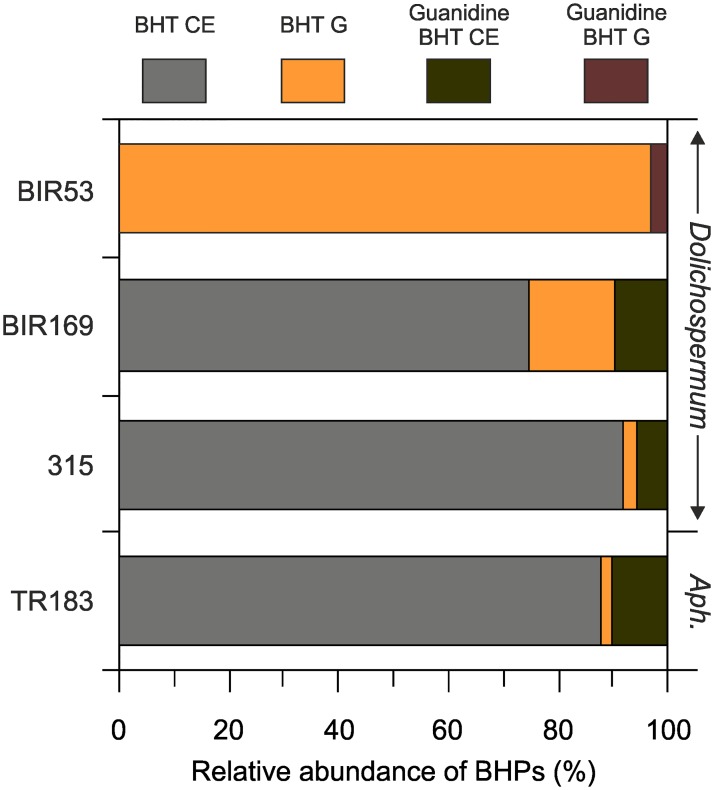
Relative abundance of bacteriohopanepolyols (BHPs) in Baltic Sea cyanobacteria. Strains 315, BIR53 and BIR169 belong to the genus *Dolichospermum*, while strain TR183 is representative of the genus *Aphanizomenon*. Note that no BHPs were detected in strains of the genus *Nodularia*. BHT CE = BHT cyclitol ether; BHT G = BHT glucosamine; guanidine BHT CE = guanidine substituted BHT cyclitol ether; guanidine BHT G = guanidine substituted BHT glucosamine.

BHP distributions were largely similar in the *Dolichospermum* and *Aphanizomenon* strains (except for *Dolichospermum* BIR53), with BHT cyclitol ether (CE) being most dominant and accounting for 74.4 to 91.6% (84.6±9.0%) of all BHPs (Fig 5; S5 Table). This component has not been reported from heterocystous cyanobacteria of the genera *Dolichospermum* and *Aphanizomenon* before, but it is common in marine and freshwater cyanobacteria, including unicellular species such as *Anacystis montana* and *Crocosphaera* sp. as well as filamentous forms like *Trichodesmium* sp. and *Chlorogloeopsis* sp. [21]. BHT CE is also among the most dominant composite BHPs found in freshwater lakes [81] and is ubiquitously distributed in temperate to tropical soils [85, 86]. Although cyanobacteria may constitute a biological source of BHT CE in these environments, it should be pointed out that this component is also synthesized by a large variety of bacterial species, including the facultative methanotroph *Methylobacterium organophilum* [87], the purple non-sulfur bacterium *Rhodoblastus acidophilus* [88], the acetic acid bacterium *Frateuria aurantia* [89] and some δ-proteobacteria belonging to the Geobacteraceae [90]. In the Baltic Sea, BHT CE (together with BHT and BHT glucosamine) has been reported from surface waters of the Gotland Deep collected during the formation of a cyanobacterial bloom dominated by *Aphanizomenon* sp. and—to a lesser extent–*Anabaena* sp. [91]. Although none of the heterocystous cyanobacteria investigated here seem to be capable of synthesizing BHT under the chosen culture conditions, the dominance of BHT CE and BHT glucosamine (see below) in *Dolichospermum* and *Aphanizomenon* is in agreement with the overall BHP distribution pattern observed during this bloom event.

Guanidine-substituted BHT CE comprised the second most abundant BHP in *Dolichospermum* 315 and BIR169 as well as in *Aphanizomenon* TR183, constituting 8.6±2.5% of all BHPs (Fig 5). Compared with BHT CE, it is less widespread in natural environments and has been reported in minor concentration only from Western Canadian soils [92] and a New Zealand geothermal vent system [93]. Guanidine-substituted BHT CE was initially described from some methylotrophic bacteria [87, 94], the α-proteobacterium *Zymomonas mobilis* [95] and more recently the δ-proteobacterium *Geobacter sulfurreducens* [90]. Given the lack of direct evidence for biological sources other than facultative and obligate anaerobes, in combination with an ubiquitous distribution of *Geobacter* in aquatic and terrestrial anoxic sediments, it has been put forward that guanidine-substituted BHT CE may be more specific for tracing *Geobacter* spp. than other BHPs, in particular when accompanied by a high amount of BHT CE [90]. Our results demonstrate, however, that BHP signatures similar to those found in *Geobacter* may also derive from cyanobacterial sources, which is in agreement with the presence of guanidine-substituted BHT CE in an enrichment culture of the unicellular cyanobacterium *Gloeocapsa* sp. [21]. As cyanobacterial biomass may be readily exported from the photic zone and incorporated into sediment sequences, care should be taken when interpreting BHP profiles in stratified aquatic systems with seasonally or permanently anoxic bottom waters and sediments.

BHT glucosamine was present in minor abundance (6.8±7.9% of all BHPs) in all *Dolichospermum* and *Aphanizomenon* strains with the exception of *Dolichospermum* BIR53 (Fig 5). This component has been described from mostly the same groups of bacteria as those producing BHT CE, including methanotrophs [87], the thermoacidophilic bacterium *Alicyclobacillus acidocaldarius* [46], the α-proteobacterium *Z*. *mobilis* [95] and two representatives belonging to *Geobacter* [90] but importantly BHT glucosamine has not been report from cultured cyanobacteria so far. It is less common in natural environments but has been reported in minor abundance from surface sediments of tropical to subtropical freshwater lakes [81] and surface water samples collected from the Baltic Sea [91]. BHT glucosamine is particularly abundant in *Dolichospermum* BIR53, in which it accounted for ca. 97% of all BHPs, making this cyanobacterium an environmentally significant source of BHT glucosamine in the Baltic Sea. *Dolichospermum* BIR53 also contained a low abundance (ca. 3%) of guanidine-substituted BHT-glucosamine. This component was reported from subsurface sediments of Lake Druzhby, Antarctica [21] but its biological source was unknown until present. Although the isolate of *Dolichospermum* BIR53 was not axenic and guanidine-substituted BHT-glucosamine may thus potentially also derive from bacterial contamination, its presence in a pure culture of *Nostoc* CCY9926 (Bauersachs, unpublished data) suggests that heterocystous cyanobacteria indeed constitute a biological source of guanidine-substituted BHT glucosamine in the Baltic Sea but also in freshwater environments.

The overall BHP distribution patterns in our Baltic Sea cyanobacteria differed markedly from those reported in the literature. BHPs commonly found to dominate the BHP distribution in pure cultures of cyanobacteria such as BHT, BHPpentol and BHP with additional C-2 methylation [21] were not detected in the heterocystous cyanobacteria investigated here. This may suggest that the restricted conditions of the Baltic, with its limited access to the open ocean and freshwater environments, may have favored the development of an endemic population of heterocystous cyanobacteria with characteristic BHP distribution patterns. Alternatively, the dramatic environmental conditions in the Baltic, with its strong salinity, temperature and nutrient gradients, may have resulted in a unique adaptation of the cyanobacterial cell membrane and the synthesis of BHPs that differ from those commonly found in freshwater and marine cyanobacteria.

### Chemotaxonomy of bloom-forming heterocystous cyanobacteria

**Fig 6:**
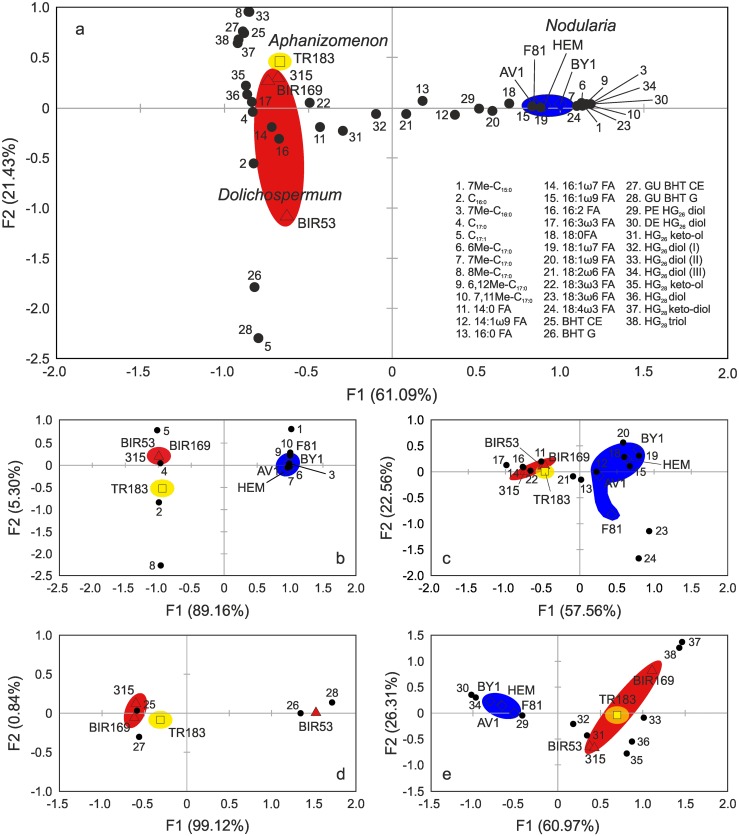
Correspondence analysis of the various lipid classes present in Baltic Sea cyanobacteria. Distribution patterns of (a) whole cell lipid distributions, (b) hydrocarbons, (c) fatty acid methyl esters, (d) bacteriohopanepolyols and (e) heterocyst glycolipids in the three genera of heterocystous cyanobacteria investigated. Dots indicate position of individual lipid biomarkers in relation to *Aphanizomenon* (square), *Dolichospermum* (triangles) and *Nodularia* (diamonds). Ellipses group the different genera of heterocystous cyanobacteria; *Aphanizomenon* (yellow), *Dolichospermum* (red) and *Nodularia* (blue).

Applying CA on whole lipid profiles (Fig 6a) and individual lipid classes (Fig 6b–6d) allowed (i) a clear separation of cyanobacteria belonging to the genus *Nodularia* from the other two genera of Baltic Sea cyanobacteria examined here and (ii) the suggestion of a close phylogenetic relationship between cyanobacteria of the genera *Dolichospermum* and *Aphanizomenon*. The distinct position of the four *Nodularia* strains in all CA plots is primarily associated with the large contributions of MMAs (6Me- and 7Me-C_17:0_), DMAs (6,12- and 7,11Me-C_17:0_), the PUFAs 18:3ω6 and 18:4ω3 as well as the heterocyst glycolipids HG_26_ diol III and deoxyhexose HG_26_ diol. These components–with the exception of 7Me-C_17:0_ occurring in minor abundance in *Aphanizomenon* TR183 –were not detected in the other heterocystous cyanobacteria investigated here and are thus considered most diagnostic for tracing planktonic cyanobacteria of the genus *Nodularia* in the Baltic Sea. Moreover, the close clustering of *Nodularia* AV1, BY1, F81 and HEM in the CA plots indicates little intraspecies variation in the lipid biomarker inventory between strains and argues for a close relationship of these heterocystous cyanobacteria. Our lipid biomarker results are thus in good agreement with molecular analyses, including whole genome [96] and 16S rRNA sequences [97], which have indicated a close overall relationship of Baltic Sea *Nodularia* strains and in fact support the finding that only one planktonic genotype of the genus *Nodularia* occurs in the Baltic Sea [80, 98].

The significant contribution of HG_28_ diol and HG_28_ keto-ol as well as HG_28_ triol and HG_28_ keto-diol, together with the presence of *n*-C_17:0_, BHT CE and guanidine-substituted BHT CE in the whole lipid CA plot, is a unique characteristic of the genera *Dolichospermum* and *Aphanizomenon* (Fig 6a). Whole lipid profiles as well as hydrocarbon, FAME and BHP distribution patterns of non-toxic *Dolichospermum* BIR169 and hepatotoxic *Dolichospermum* 315 show high overall resemblances but significantly differ in their HG content due to the exceptional high loading of HG_28_ triol and HG_28_ keto-diol to axis 2 in the CA plot (Fig 6d). The lipid inventory of BIR53 is, for the most part, similar to those in the other two *Dolichospermum* strains. Its unusual BHP signature, with the dominance of BHT glucosamine and guanidine-substituted BHT-glucosamine, however, sets it apart from other representatives of the genus *Dolichospermum* in the CA plots of both whole lipid profiles (Fig 6a) and BHP distribution patterns (Fig 6c). Although our data thus indicate that the distribution pattern of individual lipid classes may vary to some extent between the *Dolichospermum* strains, the generally close clustering of the three strains in the CA plots suggests a close overall relationship of representatives of this genus. Indeed, molecular analysis indicates that the planktonic *Dolichospermum* strains examined in this study share ≥97.8% 16S rRNA gene similarities and from a genetic perspective thus belong to the same species [32]. Similar to our results, however, the molecular data indicate a closer phylogenetic relationship between BIR169 and 315 and a more distant relationship of both strains from BIR53.

*Aphanizomenon* TR183 shows lipid signatures closely resembling those of the three *Dolichospermum* strains and it is generally associated with both the non-toxic *Dolichospermum* BIR169 and the hepatotoxic *Dolichospermum* 315 in the different CA plots (Fig 6a–6d). This similarity in lipid profiles, in particular of whole cell FAMEs (Fig 6b), BHPs (Fig 6c) and HGs (Fig 6d), suggests a close phylogenetic relationship between species of both genera, which is in contrast to the current separation of both genera based on morphological traits [99]. Previous phylogenetic studies have, however, shown that the genera *Dolichospermum* and *Aphanizomenon* are not monophyletic and occur intermixed in 16S rRNA trees [30, 71], in agreement with the lipid-based data obtained here and FAME distribution pattern reported previously [67]. Yet, there are subtle differences in the lipid biomarker inventory of *Aphanizomenon* TR183 that set it apart from all the investigated *Dolichospermum* strains. First, it is characterized by the presence of a small quantity of MMAs (such as 7Me-C_17:0_ and notably 8Me-C_17:0_), which were both absent from *Dolichospermum* spp. (Fig 1). Second, HG_26_ diol II was not observed in any of the Baltic *Dolichospermum* strains and thus is a unique characteristic of *Aphanizomenon* TR183 although it has been reported from benthic *Anabaena* CCY9613 isolated from a coastal microbial mat of the southern North Sea before [44]. Therefore, the high loading of 8Me-C_17:0_ and HG_26_ diol II onto axis 2 clearly distinguishes *Aphanizomenon* TR183 from the Baltic Sea *Dolichospermum* strains. The systematic differences in the lipid profiles of *Aphanizomenon*, *Dolichospermum* and *Nodularia* thus allow a separation of the major bloom-forming genera of heterocystous cyanobacteria and they may ultimately constitute a means of studying the community composition of cyanoHABs in the Baltic Sea and potentially other aquatic environments.

### (Paleo)environmental considerations

Blooms of N_2_ fixing heterocystous cyanobacteria have increased significantly in frequency and abundance in the Baltic Sea [4] as well as in many other brackish and freshwater environments worldwide [2]. Information on the spatiotemporal distribution and composition of cyanoHABs as well as their annual and interannual variation in modern and past ecosystems is often lacking but essential to improve our understanding on how bloom formation will proceed with changing environmental conditions (such as global warming, freshwater acidification or eutrophication) in the future. A factor that aggravates studying cyanoHABs in modern-day aquatic environments is their transitory nature and the resultant high frequency water column sampling required to adequately describe seasonal or annual variations in the intensity of cyanoHABs and their community composition. In this respect, the application of the here established lipid biomarker signatures to sediment traps, surface and subsurface sediments of the Baltic Sea (and possibly also other brackish and freshwater environments worldwide) will provide detailed information on present and past activity and diversity of bloom-forming heterocystous cyanobacteria. The time integrated nature of such records, averaging out the high seasonal and annual fluctuations of cyanoHABs, will allow obtaining a more comprehensive picture of cyanoHABs over time than can be achieved by the analysis of water column samples alone. We thus consider the here presented biomarker profiles with their high chemotaxonomic potential a valuable addition to the existing tools suited to investigate cyanoHABs in modern and moreover past ecosystems.

It has to be emphasized though that isolates stored in culture collections may adapt morphological and/or cellular traits in response to laboratory growth conditions and thereby show features departing from those found in nature, in particular when they are maintained in culture over multiple generations or over long time periods [71]. Growth conditions may also directly affect the lipid content and relative abundance of individual lipids and lipid classes as has been previously reported from a variety of algae and cyanobacteria [100, 101]. In order to minimize biases between the lipid signature of cultured isolates and natural populations of bloom-forming cyanobacteria, growth parameters (such as temperature, salinity, light intensity, etc.) were carefully chosen to match those typically encountered during the development of cyanoHABs in the Baltic Sea. In addition to culture-dependent biases, diagenetic reactions may alter the originally synthesized organic matter and not all of the established lipid signatures for *Aphanizomenon*, *Dolichospermum* and *Nodularia* may unequivocally be applied to the geological archive in order to qualitatively assess past abundances and species composition of cyanoHABs. Particularly susceptible to alteration are polyunsaturated and monounsaturated fatty acids that are readily utilized by microorganisms and in sediments show a ten times faster degradation compared to their saturated counterparts [102, 103]. Hence, fatty acid profiles such as those obtained in this study are well suited to examine bloom-forming heterocystous cyanobacteria in modern water column samples and surface sediment layers but major alterations of the originally-synthesized biological signal may be experienced over geological time scales. Information on the diagenetic stability of BHPs and HGs is currently only sparse. Both component classes have been reported from sediments of Holocene to at least Eocene age [78, 104, 105] and thus they show potential to preserve well in the sedimentary archive, in particular under strongly reducing conditions. Although additional studies on degradation rates and selective preservation of these components are needed, BHPs and HGs thus appear robust markers for the study of cyanoHABs in the Holocene Baltic Sea and likely other brackish and freshwater environments of comparable age. Normal and mid-chain branched alkanes are amongst the most refractory of organic matter and do not experience major alterations during diagenesis [106]. Given their exceptional high preservation potential with numerous reports from Middle Proterozoic to Phanerozoic sediments [107, 108] and the high specifity of mid-chain branched alkanes demonstrated in this study, these components are considered best suited to examine for the presence and community composition of cyanoHABs in the modern and past Baltic Sea and possibly also other aquatic ecosystems.

## Conclusions

Eight planktonic heterocystous cyanobacteria known to regularly from extensive cyanoHABs in the Baltic Sea were investigated for their lipid biomarker inventory. In brief, the hepatotoxic and non-toxic *Dolichospermum* strains were characterized by the presence of *n*-heptadecane and comparatively high abundances of myristic acid and BHT CE. The non-toxic *Aphanizomenon* strain showed lipid distributions very similar to those of *Dolichospermum* spp. but also contained HG_26_ diol II, MMAs (including 7Me-C_17:0_ and 8Me-C_17:0_) in minor proportion and lacked a higher relative abundance of myristic acid. In contrast, all *Nodularia* strains were recognized as important sources of MMAs (6Me-C_17:0_ and 7Me-C_17:0_) and DMAs (6,12Me-C_17:0_ and 7,11Me-C_17:0_). They also contained HG_26_ diol III, deoxyhexose HG_26_ diol and the PUFAs 18:3ω6 and 18:4ω3, which were absent from the other heterocystous cyanobacteria investigated. These differences in lipid distributions support the current taxonomic separation of bloom-forming heterocystous cyanobacteria into three distinct genera but they also indicate a close phylogenetic relationship between species of the genera *Dolichospermum* and *Aphanizomenon*. Compared with the classical biomarker approach, in which a single component or a component class is employed to trace for a certain group of organism, the analysis of multiple lipid classes in water column samples, surface and subsurface sediments will allow a more detailed reconstruction of the community composition of cyanoHABs in the Baltic Sea and their variation over time. In addition, our study highlights the importance of dedicated culture studies to unequivocally link biomarker profiles found in the geosphere to their biological sources and thus improve our understanding on the spatiotemporal distribution of microbial communities (such as bloom-forming cyanobacteria) and their response to environmental changes in modern and fossil ecosystems.

## Supporting information

S1 Table Cyanobacterial strains and culture conditions. (XLSX)

S2 Table Relative distribution of straight and branched hydrocarbons. (XLSX)

S3 Table Relative distribution of fatty acid methyl esters. (XLSX)

S4 Table Relative distribution of heterocyst glycolipids. (XLSX)

S5 Table Relative distribution of bacteriohopanepolyols. (XLSX)
